# A study on the relationship between mMRC dyspnea scale and both risk stratification and poor prognosis in patients with acute pulmonary embolism

**DOI:** 10.3389/fcvm.2026.1706804

**Published:** 2026-04-28

**Authors:** Yulang Xiong, Chengwei Liu

**Affiliations:** Department of Cardiology, Wuhan Asia Heart Hospital Affiliated with Wuhan University of Science and Technology, Wuhan, China

**Keywords:** acute pulmonary embolism, classification of pulmonary embolism severity, modified Medical Research Council dyspnea scale, poor prognosis, risk stratification

## Abstract

**Background:**

This study aimed to investigate the value of the modified Medical Research Council (mMRC) dyspnea scale in risk stratification and outcome assessment for patients with acute pulmonary embolism (APE).

**Methods:**

A retrospective analysis was performed using medical records from a tertiary care center between 2011 and 2023. The study included patients aged 18–80 years who were diagnosed with APE. Participants were categorized into groups based on pulmonary embolism risk stratification, mMRC dyspnea scale, and the presence or absence of adverse outcomes within 1 year, which included in-hospital mortality, all-cause mortality after discharge, and hospital readmission. The associations between the mMRC dyspnea scale and both APE risk stratification and 1-year adverse outcomes were evaluated. The predictive performance of the mMRC dyspnea scale for 1-year adverse prognosis was assessed using receiver operating characteristic (ROC) curve analysis to determine the optimal cut-off threshold.

**Results:**

The study demonstrated that the mMRC dyspnea scale was significantly positively correlated with risk stratification of APE (*P* < 0.05). Moreover, the Qanadli score, systolic blood pressure, cardiac troponin, N-terminal pro-brain natriuretic peptide, and the right-to-left ventricular ratio were significantly associated with higher mMRC scores (*P* < 0.05). ROC curve analysis revealed that an mMRC dyspnea grade of 3 was considered the optimal cut-off value for predicting adverse prognosis within 1 year, with an area under the curve of 0.803 (*P* < 0.001).

**Conclusion:**

The mMRC dyspnea scale demonstrates a significant association with risk stratification in patients with APE. An mMRC dyspnea grade of ≥3 is indicative of a higher risk for adverse outcomes within 1 year and may serve as a valuable prognostic indicator for predicting clinical outcomes in APE patients.

## 1 Introduction

Pulmonary embolism (PE) is a pathological condition or syndrome resulting from the obstruction of the pulmonary arterial circulation by various types of emboli (1). It affects approximately 10 million individuals globally each year (2). In China, the annual hospitalization rate for PE increased from 0.012‰ in 2007 to 0.071‰ in 2016, while the in-hospital mortality rate declined from 8.5% to 3.9%. Recent studies indicated that the incidence of acute pulmonary embolism (APE) among hospitalized patients in China reached 0.14‰ in 2021. Furthermore, the APE-related mortality rate in China now aligns with that observed in Europe, North America, and other Asian countries, demonstrating a marked downward trend; by 2021, the in-hospital mortality rate among APE patients had decreased to 0.1‰. Despite this decline, PE continues to be associated with considerable mortality (3, 4). Therefore, precise evaluation of disease severity and short-term prognosis in APE patients is crucial for the optimization of therapeutic strategies (5). Dyspnea represents one of the most frequently encountered clinical manifestations in APE patients. The modified Medical Research Council (mMRC) dyspnea scale offers a standardized method for quantifying the severity of breathlessness, thereby serving as a valuable tool in clinical assessment, particularly for respiratory disorders. While existing research has primarily focused on the association between mMRC dyspnea score, patient quality of life, and exercise capacity, there remains limited evidence regarding its correlation with risk stratification and adverse clinical outcomes in APE. Hence, this study sought to investigate the clinical utility of the mMRC dyspnea scale in the management of APE patients.

## 2 Materials and methods

### 2.1 Selection and grouping

This retrospective, non-randomized, single-center study was conducted at a tertiary care center. Among 585 patients diagnosed with PE, 327 cases of APE were identified after excluding 45 patients who did not meet the inclusion criteria. A total of 282 APE patients were categorized into two groups based on their mMRC scores and risk stratification for adverse outcomes. Data for all enrolled patients were extracted from medical records and evaluated independently by two clinical physicians. Any discrepancies in assessments between the two physicians were resolved by a third researcher. Short-term mortality data and hospital readmission rates were obtained through telephone follow-ups and patient discharge records.

### 2.2 Inclusion criteria

The 200 patients included in this study were all diagnosed upon admission by computed tomography angiography (CTPA) (enhanced scanning of the pulmonary artery using a 64-slice spiral CT machine produced by Philips, Suzhou, China). The patients also meet the following criteria:

1. duration of the patient's clinical symptoms ≤14 days;
2. patients aged between 18 and 80 years; and
3. patients required to have a body mass index (BMI) < 30 kg/m^2^ (to exclude the confounding effects of obesity on dyspnea).

### 2.3 Exclusion criteria

To minimize confounding factors and more clearly elucidate the relationship between APE and the mMRC dyspnea scale, the following patients were excluded:

1. patients with chronic dyspnea (mMRC dyspnea scale grades ≥1 in the past 6 months) whose symptoms were not due to newly developed respiratory diseases such as bronchial asthma, chronic obstructive pulmonary disease, interstitial pneumonia, or chronic post-embolic pulmonary hypertension;
2. patients with circular system diseases such as chronic heart failure, valvular heart disease, dilated cardiomyopathy, hypertrophic cardiomyopathy, and restrictive cardiomyopathy that lead to exertional dyspnea;
3. duration of the patient's clinical symptoms >14 days;
4. patients aged <18 or >80 years; and
5. patients with BMI ≥30 kg/m^2^.

### 2.4 Assessment of mMRC dyspnea scale

See Table 1 for details about the mMRC dyspnea scale.

**Table 1:** mMRC dyspnea scale.

mMRC grade	Dyspnea symptoms	
Grade 0	I only get breathless with strenuous exercise.	Running exercise, heavy physical chores (moving, thorough, cleaning), fieldwork, climbing more than 5 flights of stairs.
Grade 1	I get short of breath when hurrying on a level or walking up a slight hill.	Long-distance walking, fast walking, climbing small hills, going up stairs (3–4 floors), decreased tolerance for physical activities.
Grade 2	I walk slower than people of the same age on a level because of breathlessness, or I have to stop for breath when walking at my own pace on a level.	Leisurely walking, going up stairs (1–2 floors), bowel movements, mopping the floor, doing laundry, cooking, arguing.
Grade 3	I stop for breath after walking about 100 meters or after a few minutes on a level.	Light household chores (washing vegetables, wiping tables), getting dressed, personal hygiene (washing up), urinating, speaking quickly.
Grade 4	I am too breathless to leave the house or I am breathless when dressing or undressing.	Sleeping and resting, speaking normally, moving around.

### 2.5 Risk stratification for patients with APE

Risk stratification of APE patients was performed according to the 2019 European Society of Cardiology (ESC) Guidelines for the diagnosis and management of acute pulmonary embolism, developed in collaboration with the European Respiratory Society (ERS) (Table 2).

**Table 2:** Risk stratification of APE.

Early mortality risk		Indicators of risk			
		Hemodynamic instability	PESI class III–V or sPESI ≥1	RV dysfunction on TTE or CTPA	Elevated cardiac troponin levels
	High	+	+	+	+
Intermediate	Intermediate–high	−	+	+	+
	Intermediate–low	−	+	One (or none) positive	
	Low	−	−	−	−

PESI, pulmonary embolism severity index; sPESI, simplified pulmonary embolism severity index; TTE, transthoracic echocardiography.

### 2.6 Observation indicators

1. The mMRC dyspnea scale assessed at admission was extracted from the medical records of patients. Risk stratification of APE patients was evaluated according to the 2019 ESC Guidelines for the diagnosis and management of acute pulmonary embolism, developed in collaboration with the ERS.
2. Laboratory test data after admission included the patient's cardiac troponin (cTnI) and N-terminal pro-brain natriuretic peptide (NT-proBNP).
3. Baseline data of the patients included age, gender, systolic blood pressure (SBP) at admission, diabetes, hypertension, hyperlipidemia, deep vein thrombosis (DVT) or varicose veins, coronary heart disease, and smoking status.
4. Imaging data: The direct diagnostic sign of APE via CTPA findings is the presence of low-density filling defects within the pulmonary artery. The pulmonary embolism index is calculated using the Qanadli method, with assessments performed collaboratively by at least two radiologists with more than 10 years of experience in CT diagnostics. The right/left ventricular (RV/LV) ratio is also measured by CTPA.
5. Adverse prognosis of patients within 1 year: Mortality and hospital readmission status of all patients within 1 year were determined through telephone follow-up or patient revisit records and included in the study.

### 2.7 Statistical methods

Statistical analysis was performed using SPSS 27 for windows. Normally distributed data are expressed as mean ± standard deviation, skewed data are expressed as median (upper quartile–lower quartile), and categorical variables are expressed as frequency (%). Multiclass normally distributed data were tested using one-way ANOVA, binary normally distributed data were tested using independent samples t-tests, multiclass non-normally distributed data were tested using K independent samples, binary non-normally distributed data were tested using Mann–Whitney *U* tests, differences in categorical variables were assessed using chi-square tests, and the correlation between ordinal variables was tested using Spearman correlation analysis. ROC curve analysis was conducted to determine the optimal cut-off value of mMRC for predicting adverse prognosis at 1 year. A *p*-value < 0.05 was considered statistically significant.

## 3 Results

### 3.1 Baseline characteristics of APE patients across all mMRC dyspnea scale grades (0–4)

The baseline characteristics of APE patients showed no significant differences in age, gender, prevalence of hypertension, coronary heart disease, diabetes, hyperlipidemia, DVT or varicose veins, and smoking rates among the five mMRC dyspnea groups from 0 to 4 (*p-*values were 0.86, 0.14, 0.44, 0.36, 0.31, 0.67, 0.40, and 0.23, respectively) (Table 3).

**Table 3:** Baseline characteristics of APE patients in the mMRC dyspnea scale grades of 0–4.

Characteristics	mMRC dyspnea scale grades						
	0 (*n* = 29)	1 (*n* = 48)	2 (*n* = 55)	3 (*n* = 99)	4 (*n* = 51)	*χ*^2^/F	*P*
Age (years)	64.10 ± 12.74	66.56 ± 8.32	65.67 ± 9.50	65.07 ± 11.53	64.53 ± 12.14	0.33	0.86
Gender, *n* (%)						6.90	0.14
Male	7 (24.10)	15 (31.30)	23 (41.80)	37 (37.40)	11 (21.60)		
Female	22 (75.90)	33 (68.80)	32 (58.20)	62 (62.60)	40 (78.40)		
Hypertension, *n* (%)	20 (69.00)	30 (62.50)	27 (49.10)	56 (56.60)	28 (54.90)	3.79	0.44
Coronary heart disease, *n* (%)	4 (13.80)	3 (6.30)	2 (3.60)	5 (5.10)	2 (3.90)	4.39	0.36
Diabetes, *n* (%)	1 (3.40)	7 (14.60)	9 (16.40)	8 (8.10)	6 (11.80)	4.83	0.31
Hyperlipidemia, *n* (%)	4 (13.80)	8 (16.70)	5 (9.10)	12 (12.10)	4 (7.80)	2.38	0.67
DVT or varicose veins, *n* (%)	4 (13.80)	3 (6.30)	7 (12.70)	12 (12.20)	10 (19.60)	4.01	0.40
Smoking, *n* (%)	6 (20.70)	4 (8.30)	9 (16.40)	15 (15.20)	3 (5.90)	5.63	0.23

DVT, deep vein thrombosis.

### 3.2 Poor prognosis in APE patients across all mMRC dyspnea scale grades (0–4)

Analysis of adverse prognosis across mMRC dyspnea scale grades in patients with APE demonstrated statistically significant differences in outcomes—including in-hospital mortality, 1-year post-discharge mortality, and 1-year readmission rates (all *P* < 0.05) (Table 4).

**Table 4:** Poor prognosis in patients with APE across all mMRC dyspnea scale grades (0–4).

Poor prognosis	mMRC dyspnea scale grades						
	0 (*n* = 29)	1 (*n* = 48)	2 (*n* = 55)	3 (*n* = 99)	4 (*n* = 51)	χ^2^/F	*P*
In-hospital mortality, *n* (%)	0 (0)	0 (0)	0 (0)	3 (3.0)	5 (9.8)	12.8	0.012
Mortality within 1 year post-discharge, *n* (%)	0 (0)	1 (2.1)	1 (1.8)	13 (13.1)	9 (17.6)	16.59	0.002
1-year readmission rate, *n* (%)	0 (0)	0 (0)	1 (1.8)	18 (18.2)	8 (15.7)	22.65	<0.001

### 3.3 Risk stratification and mMRC dyspnea scale contingency for patients with APE

Table 5 shows that 89.72% of all included APE patients experienced dyspnea. Among the 45 low-risk patients, 29 (64.44%) had dyspnea (mMRC scale ≥ 1). The prevalence rates of dyspnea in the low-to-moderate risk, moderate-to-high risk, and high-risk groups were 80.88% (55 out of 68), 100% (121 out of 121), and 100% (48 out of 48), respectively. Spearman's rank correlation analysis revealed a significant positive correlation between mMRC dyspnea scale grades and APE risk stratification (*r* = 0.70, *P* < 0.05), indicating that higher risk stratification levels in APE are associated with increased severity of dyspnea (Table 5).

**Table 5:** Risk stratification and mMRC dyspnea scale contingency table for patients with APE.

mMRC score		Risk stratification of APE			
		High	Intermediate–high	Intermediate–low	Low
mMRC score	0	0 (0%)	0 (0%)	13 (19.12%)	16 (35.56%)
	1	2 (4.17%)	1 (0.83%)	22 (32.35%)	23 (51.11%)
	2	8 (16.67%)	17 (14.05%)	25 (36.76%)	5 (11.11%)
	3	22 (45.83%)	70 (57.85%)	6 (8.82%)	1 (2.22%)
	4	16 (33.33%)	33 (27.27%)	2 (2.94%)	0 (0%)
	Total	48 (17.02%)	121 (42.91%)	68 (24.11%)	45 (15.96%)

**Table 6:** The key biochemical and imaging parameters associated with risk stratification of APE patients across different mMRC dyspnea scale grades.

Characteristics	mMRC dyspnea scale grades						
	0 (*n* = 29)	1 (*n* = 48)	2 (*n* = 55)	3 (*n* = 99)	4 (*n* = 51)	F/H	*P*
cTnI	0.012 (0.006–0.018)	0.013 (0.006–0.022)	0.059 (0.020–0.15)	0.087 (0.034–0.18)	0.11 (0.044–0.20)	77.47	<0.001
NT-proBNP	226.40 (78.87–327.55)	245.80 (157.18–835.65)	948.00 (238.60–2,561.00)	1,102.00 (420.90–2,986.00)	1,813.00 (678.40–3,906.00)	49.32	<0.001
RV/LV ratio	0.83 ± 0.23	0.93 ± 0.21	1.11 ± 0.35	1.43 ± 0.39	1.62 ± 0.42	44.20	<0.001
SBP	132.48 ± 15.69	136.96 ± 24.46	129.25 ± 21.85	128.93 ± 26.40	105.55 ± 14.48	14.72	<0.001
PE index	37.50 (26.50–42.00)	44.00 (33.75–59.63)	57.50 (39.00–65.50)	70.00 (52.50–77.50)	75.00 (67.50–82.50)	98.97	<0.001

cTnI, cardiac troponin I; NT-proBNP, N-terminal pro b-type natriuretic peptide.

### 3.4 Key biochemical and imaging parameters associated with risk stratification of APE patients across different mMRC dyspnea scale grades

**Figure 2:**
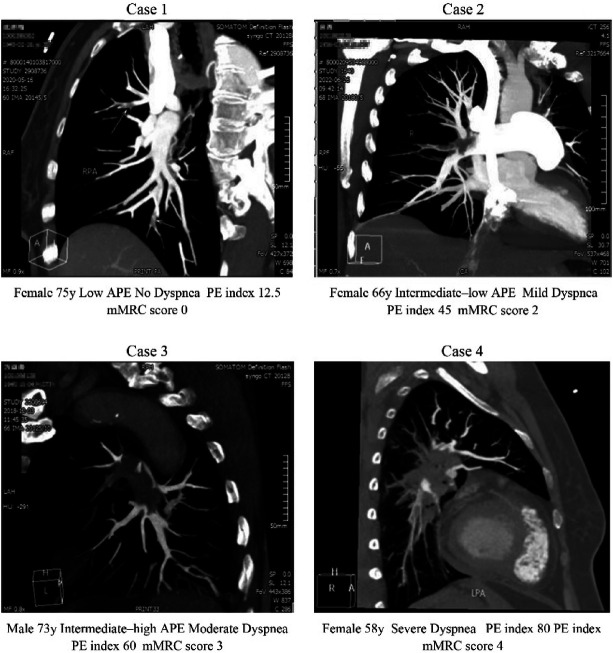
ROC curve of mMRC dyspnea scale grades and 1-year adverse prognosis in APE patients.

Grouping of APE patients by mMRC dyspnea scale included 29, 48, 55, 99, and 51 patients in mMRC dyspnea grades 0, 1, 2, 3, and 4, respectively. Significant differences were observed across groups in cardiac cTnI, NT-proBNP, RV/LV ratio, SBP, and pulmonary embolism index (*p-*values are <0.001 for each) (Table 6, Figure 2).

### 3.5 Comparison of prognostic-related clinical indicators between the two groups with and without adverse prognosis

APE patients were divided into two groups based on whether or not they experienced adverse prognosis within 1 year. Independent samples t-tests showed that there were significant differences in systolic blood pressure, length of hospitalization, pulmonary embolism index, right-to-left ventricle ratio, NT-proBNP, and cTnI between the two groups (*p*-values are <0.001 for each) (Table 7).

**Table 7:** Comparison of prognostic-related clinical indicators between the two: groups with and without adverse prognosis.

Characteristics	No poor prognosis group (*n* = 254)	Poor prognosis group (*n* = 65)	*t*/*z*	*P*
SBP	131.94 ± 21.75	105.92 ± 23.71	0.25	<0.001
PE index	57.50 (42.50–70.00)	72.00 (60.00–80.00)	−4.90	<0.001
RV/LV ratio	1.18 ± 0.41	1.53 ± 0.47	1.35	<0.001
NT-proBNP	678.40 (213.70–2,320.00)	1,610.00 (461.20–3,062.00)	−2.75	0.006
cTnI	0.035 (0.012–0.12)	0.14 (0.068–0.31)	−6.15	<0.001

cTnI, cardiac troponin I; NT-proBNP, N-terminal pro b-type natriuretic peptide.

### 3.6 ROC curve of mMRC dyspnea scale and 1-year adverse prognosis in APE patients

**Figure 1:**
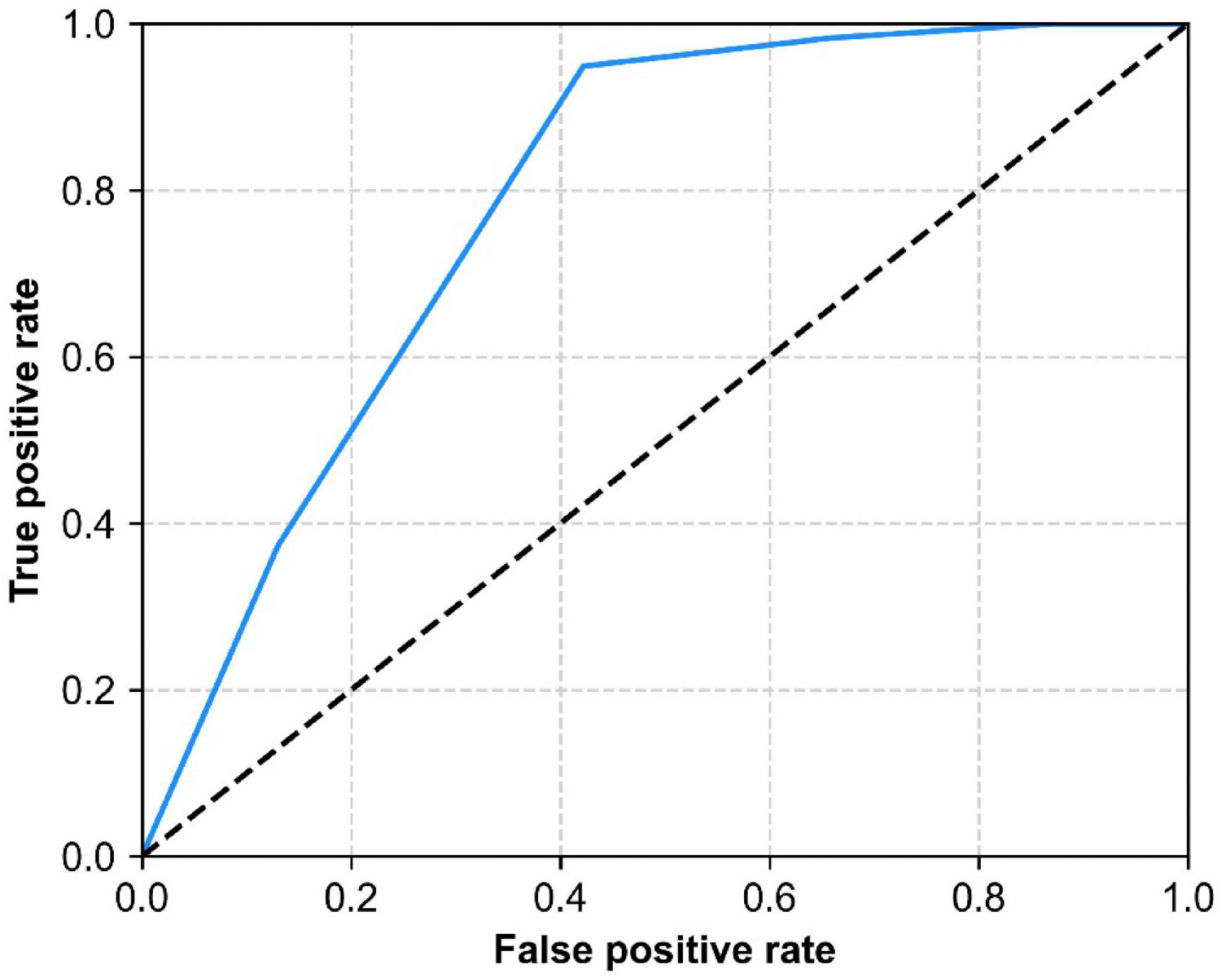
CTPA.

The ROC curve analysis demonstrated an area under the curve of 0.803, with a significance level of less than 0.001, indicating a significant correlation between the mMRC dyspnea scale and 1-year adverse prognosis in APE patients. The sensitivity was 94.9% and the specificity was 57.8%. The Youden index was 0.528, corresponding to an mMRC dyspnea grade of 2.5. As the mMRC dyspnea scale is graded as an ordinal variable, an mMRC grade of 3 was considered the optimal cut-off point for the ROC curve. An mMRC dyspnea grade ≥3 was associated with adverse prognosis in APE patients within 1 year (Figure 1).

## 4 Discussion

PE ranks third in mortality among cardiovascular diseases (6). Its clinical presentation is highly variable (7), contributing to both missed and incorrect diagnoses in clinical practice. Dyspnea represents the most frequently observed symptom in PE patients, affecting approximately 80%–90% of patients (1, 2, 7, 8). Within the classical triad of PE symptoms, dyspnea remains the most prevalent. Therefore, investigating the association between dyspnea severity and both risk stratification and prognostic outcomes in APE holds significant clinical relevance. However, dyspnea exhibits relatively low specificity and poses challenges in objective quantification. The mMRC dyspnea scale provides a standardized tool for evaluating and quantifying the severity of breathlessness, primarily utilized in the context of respiratory disorders. To date, research on the mMRC dyspnea scale in APE has predominantly focused on its relationship with quality of life and exercise capacity in PE patients, while studies examining its correlation with risk stratification and adverse clinical outcomes remain limited (9). In this study, we investigated the relationship between mMRC dyspnea scale and both risk stratification and adverse prognosis in APE patients, with the aim of offering novel insights into the early identification of high-risk individuals and the optimization of therapeutic decision-making.

Existing evidence demonstrates a strong correlation between the pulmonary embolism index and the risk stratification in patients with PE (10). Qanadli et al. developed the CTPA-based pulmonary embolism index by assessing the location of pulmonary artery emboli, which has been validated as predictive of APE severity (8). In our study, cases with higher patient risk stratifications—such as patients 1 and 4—exhibited elevated pulmonary embolism indices and higher mMRC dyspnea scale grades. According to the 2019 ESC Guidelines for the Diagnosis and Management of APE, developed in collaboration with the ERS, APE risk stratification relies on a comprehensive evaluation of hemodynamic status, myocardial injury markers, and right ventricular function (1). SBP serves as a critical early indicator for APE risk stratification and effectively distinguishes high-risk from intermediate-risk patients. Hemodynamic abnormalities typically occur when 30% of the pulmonary arterial bed is obstructed by thrombotic material, a key factor contributing to APE onset (11). A right ventricular-to-left ventricular diameter ratio of ≥0.9 or 1 indicates right ventricular dysfunction (RVD). A marker for myocardial injury, elevation of cTnI in APE with RVD reflects myocardial damage, with higher levels indicating greater severity. Similarly, NT-proBNP is a cardiac hormone secreted by ventricular myocytes in response to ventricular dilation or increased pressure load. In APE patients, an increase in NT-proBNP correlates with heightened right ventricular afterload and wall tension, reflecting the severity of RVD and hemodynamic instability. Elevated NT-proBNP levels in patients without established cardiac disease should prompt consideration of pulmonary embolism.

Determining the risk of adverse outcomes in patients is essential for guiding treatment decisions for individuals with APE (12). Meta-analyses have demonstrated that, compared to patients without right ventricular dysfunction, those with higher RV/LV ratios as defined by CT imaging exhibit significantly increased mortality risks (13). Moreover, studies have confirmed that the RV/LV ratio and pulmonary artery obstruction index can accurately and non-invasively predict adverse clinical outcomes in APE patients (14). Various biomarkers—including cTnI and NT-proBNP—have been extensively evaluated in cohort studies and meta-analyses. These biomarkers identify populations at risk of poor prognosis through one or more clinical endpoints and are valuable tools for predicting adverse events in APE patients. However, the optimal selection of biomarkers and their ideal cut-off values remain subjects of debate. In terms of short-term adverse outcomes, elevated levels of cTnI are associated with increased short-term all-cause mortality or poor prognosis, while elevated levels of NT-proBNP also indicate a higher likelihood of short-term adverse events (15, 16). Regarding long-term adverse outcomes, two studies have suggested an association between NT-proBNP levels and long-term adverse prognosis; however, these studies were limited by small sample sizes, necessitating further investigation with larger cohorts to confirm these findings. There is currently no statistically significant evidence supporting the long-term prognostic value of cTnI (17). Cozzi et al. reported that the pulmonary embolism index and NT-proBNP levels correlate with adverse outcomes within 1 year following PE diagnosis (18). To date, no studies have specifically examined the relationship between systolic blood pressure and one-year adverse outcomes in APE patients. The mMRC dyspnea scale is closely linked to multiple health status indicators (19) and serves as a predictor of future mortality risk. Nobuhiro Asai et al. identified an mMRC dyspnea grade of ≥3 as a poor prognostic factor in chronic obstructive pulmonary disease (COPD) (20), while Poulos et al. found that an mMRC dyspnea grade of ≥2 correlates with adverse outcomes in patients with cardiopulmonary diseases accompanied by dyspnea (19).

Through this study, we demonstrated that the mMRC dyspnea scale is associated with risk stratification in APE. An mMRC dyspnea grade of ≥3 represents a critical threshold for mortality risk in APE patients and correlates with poor prognosis within 1 year. Moreover, we identified that cTnI, NT-proBNP, RV/LV ratios, and SBP are independently associated with one-year adverse outcomes in APE patients. PE risk stratification, which is based on short-term mortality risk, facilitates initial treatment decisions (21) and has been shown to reduce hospitalization duration and PE-related mortality (22, 23). The results of this study indicate that there is a significant correlation between the mMRC dyspnea scale and the risk stratification of patients with APE. Patients with an mMRC dyspnea scale of ≥3 are at high risk of poor prognosis within 1 year, suggesting that the mMRC dyspnea scale may serve as a simple, safe, and useful indicator for predicting the prognosis of APE patients. In clinical practice, the mMRC dyspnea scale may assist clinicians in quickly and concisely assessing the severity of patient conditions in emergency situations, enabling critically ill patients or those at potential risk of deterioration to receive timely treatment. In addition, we can also consider applying the results of this study to health education, enabling non-medical personnel to simply and rapidly assess severe dyspnea, thus gaining more time for diagnosis and treatment of patients after admission.

This study has some limitations. As a retrospective, non-randomized, single-center cohort study, the sample size was relatively small. In particular, the limited number of endpoint events and incomplete clinical data prevented the use of multivariate analysis, which restricted us from obtaining more convincing results. Furthermore, the exclusion of obese patients and those with chronic cardiopulmonary diseases limits the direct application of the research findings to a broader and more general population of APE patients, especially those with common cardiopulmonary diseases or obesity. In the future, large-scale, multicenter, randomized controlled trials are required to further explore the correlation between the mMRC dyspnea scale and risk stratification and adverse prognosis in APE patients.

## 5 Conclusion

The mMRC dyspnea scale demonstrates a significant association with risk stratification in patients with APE. An mMRC dyspnea grade ≥3 is indicative of a higher risk for adverse outcomes within 1 year and may serve as a valuable prognostic indicator for predicting clinical outcomes in APE patients.

## Data Availability

The raw data supporting the conclusions of this article will be made available by the authors without undue reservation.
